# Predictive value of contrast-enhanced MRI for the regrowth of residual uterine fibroids after high-intensity focused ultrasound treatment

**DOI:** 10.1186/s13244-024-01839-w

**Published:** 2024-11-15

**Authors:** Yang Liu, Zhibo Xiao, Yuanli Luo, Xueke Qiu, Lu Wang, Jinghe Deng, Mengchu Yang, Fajin Lv

**Affiliations:** 1 The State Key Laboratory of Ultrasound in Medicine and Engineering, College of Biomedical Engineering, Chongqing Medical University, Chongqing, 400016 China; 2 Department of Radiology, The First Affiliated Hospital of Chongqing Medical University, Chongqing, China; 3 Present Address: Department of Radiology, The First Affiliated Hospital of Chongqing Medical University, Chongqing, China

**Keywords:** Residual uterine fibroids, High-intensity focused ultrasound, Contrast-enhanced MRI, T2-weighted image, Quantitative analysis

## Abstract

**Objectives:**

To investigate whether the signal intensity (SI) ratio of residual fibroid (RF) to myometrium using Contrast-Enhanced Magnetic Resonance Imaging (CE-MRI) could predict fibroid regrowth after high-intensity focused ultrasound (HIFU) treatment.

**Materials and methods:**

A retrospective analysis was conducted among 164 patients with uterine fibroids who underwent HIFU. To predict the RF regrowth, the SI perfusion parameters were quantified using the RF-myometrium SI ratio on CE-MRI on day 1 post-HIFU and then compared with the fibroid-myometrium SI ratio on the T2-weighted image (T2WI) and Funaki classification 1 year later. Thirty cases from another center were used as an external validation set to evaluate the performance of RF-myometrium SI ratio.

**Results:**

The predictive performance of the RF-myometrium SI ratio on CE-MRI on day 1 post-HIFU (Area Under Curve, AUC: 0.869) was superior to that of the preoperative and postoperative fibroid-myometrium SI ratios on the T2WI (AUC: 0.724, 0.696) and Funaki classification (AUC: 0.663, 0.623). Multivariate analysis showed that the RF- myometrium SI ratio and RF thickness were independent factors. The RF-myometrium SI ratio reflects the long-term rate of re-intervention (*r* = 0.455, *p* < 0.001).

**Conclusion:**

The RF-myometrium SI ratio on CE-MRI exhibits greater accuracy in predicting RF regrowth compared to the SI classification and the SI ratio on T2WI.

**Critical relevance statement:**

The ratio of residual uterine fibroid to myometrial signal intensity on contrast-enhanced (CE)-MRI can reflect residual blood supply, predict regrowth of fibroids, and thus reflect long-term re-intervention rate and recovery situation of clinical high-intensity focused ultrasound (HIFU) treatment.

**Key Points:**

- Contrast-enhanced (CE)-MRI can indicate the blood supply of residual uterine fibroids after high-intensity focused ultrasound (HIFU) treatment.
- The predictive capability of CE-MRI ratio surpasses T2WI ratio and the Funaki.
- Residual fibroids can serve as a measure of the long-term efficacy of HIFU.

**Graphical Abstract:**

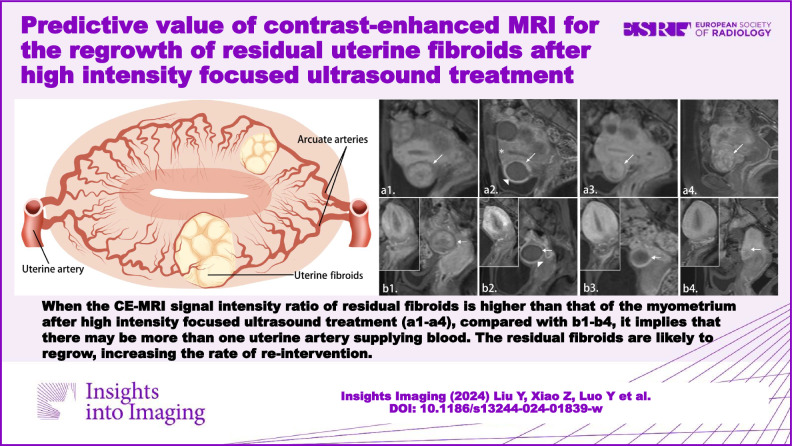

## Introduction

Uterine fibroids are monoclonal tumors originating from the uterine smooth muscle [1, 2]. Nearly half of the patients with uterine fibroids presented various symptoms, such as menorrhagia, mass-related pressure symptoms, and reproductive dysfunction, seriously affecting their quality of life [3]. High-intensity focused ultrasound (HIFU) is a safe and effective technology and is widely used in patients with uterine fibroids [4, 5]. Previous studies have shown that 70.1–84.6% of patients needed re-interventions after HIFU treatment because of an increase in fibroid volume [6–8]. In fact, the regeneration of fibroids is the regrowth of residual fibroids (RFs) [9, 10]. Although an ablation is deemed successful when the non-perfusion volume ratio (NPVR) ≥ 80% [11, 12], RFs may have a chance to regrow. To avoid long-term re-intervention and relieve the symptoms, it is highly essential to predict whether RFs would regenerate after HIFU treatment.

In Digital Subtraction Angiography (DSA) examinations among patients undergoing uterine artery embolization (UAE) for the treatment of uterine fibroids, variations in blood supply among different fibroids can lead to different prognoses. The imaging of fibroids supplied by bilateral uterine arteries appears earlier than that of myometrium, which might affect the regrowth of fibroids [10, 13, 14]. In HIFU treatment, DSA examination is not as convenient as Magnetic Resonance Imaging (MRI), and the application range is relatively small [5, 15].

MRI, with a high resolution for soft tissue, is an important method for the evaluation of HIFU treatment for uterine fibroids. In the past, the signal intensity (SI) classification or ratio relationship of T2-weighted image (T2WI) and contrast-enhanced MRI (CE-MRI) is the main parameter of MRI in the evaluation of HIFU treatment for uterine fibroids [16, 17]. Compared to the T2WI, the CE-MRI can directly reflect the blood perfusion inside the tumor, and RFs can be observed [18, 19]. Currently, few studies focus on whether the SI ratio of RF to myometrium by CE-MRI can indicate the internal blood supply of the RF and their implications.

In this study, we aim to quantify whether the SI ratio of CE-MRI RF-myometrium, reflecting the blood supply within RFs after HIFU treatment, can be used to predict their regrowth, compared to the Funaki classification and SI ratio from the T2WI.

## Materials and methods

This retrospective study was approved by the Ethics Committee of our institution (Approval No. HF2023-011); the requirement for informed consent was waived. All procedures adhered to the hospital’s ethical standards and the principles of the Declaration of Helsinki.

### Patients

We included patients who presented to Chongqing Haifu Hospital and were treated with ultrasound-guided HIFU for uterine fibroids between January 2016 and December 2021.

Patients were eligible in this study if they have: (1) older than 18 years old and before menopausal; (2) with complete data of MRI examination before, after, and 1 year after treatment (9–17 months); (3) without surgical history for uterine fibroid before HIFU treatment; (4) under a single-session HIFU treatment. The exclusion criteria were: (1) low-quality MR imaging or incomplete data; (2) contraindications for MRI examination; (3) suspected malignant uterine tumors; and (4) uterine fibroids with obvious spontaneous necrosis, calcification, or degeneration before treatment and postoperative bleeding suggested by MRI.

### MRI examination and HIFU treatment

All patients underwent 1.5-T MR (uMR570, China United Imaging Company) using standard T2WI and CE-MRI; the acquisition parameters are listed in Table 1. A JC200 or JC-high-intensity focused ultrasound system (Chongqing Haifu Medical Technology Co., Ltd., Chongqing, China) was used for tumor treatment.

**Table 1:** MRI scan parameters

MRI type	Repetition time (ms)	Echo time (ms)	Number of excitations	Field of view (cm × cm)	Matrix size (mm × mm)	Slice thickness (mm)	Slice gap (mm)	Imaging planes
T2-weighted	5300/4060	88/88	2/3	24 × 24/22.4 × 28	320 × 75/288 × 224	5/5	1/1.5	T, S
CE-MRI	3.94/4.2	1.84/1.9	1/0.72	35 × 28/38 × 30.4	288 × 75/320 × 224	4/2.5	0.5/0	T, S, C

The parameters used for MRI are illustrated in the table

*C* coronal plane, *S* sagittal plane, *T* transverse plane

### Clinical data and MRI features, criteria for RF regrowth

The patient’s age, BMI, menstrual disorder, number of pregnancies and births, age at menarche, menstruation, menstrual cycle, menstrual volume, history of smoking and drinking were collected, respectively.

Two radiologists with 6 years of pelvic radiation experience and a gynecologist with 16 years of clinical experience performed the evaluations together. Residual fibroids (RFs) are defined based on the degree of enhancement observed on CE-MRI following HIFU treatment. The internal SI ratio of the uterine fibroid is calculated using the following formula [9]: Internal SI ratio = (post-CE-MRI SI of the fibroid / post-CE-MRI SI of the iliacus muscle) / (pre-CE-MRI SI of the fibroid / pre-CE-MRI SI of the iliacus muscle). If the internal SI ratio of the fibroid is greater than 1%, it indicates persistent enhancement post-operatively, defining the fibroid as residual. Conversely, if the internal SI ratio is less than or equal to 1%, it indicates minimal to no enhancement, categorizing the area as Non-Perfusion Volume (NPV).

**Fig. 1:**
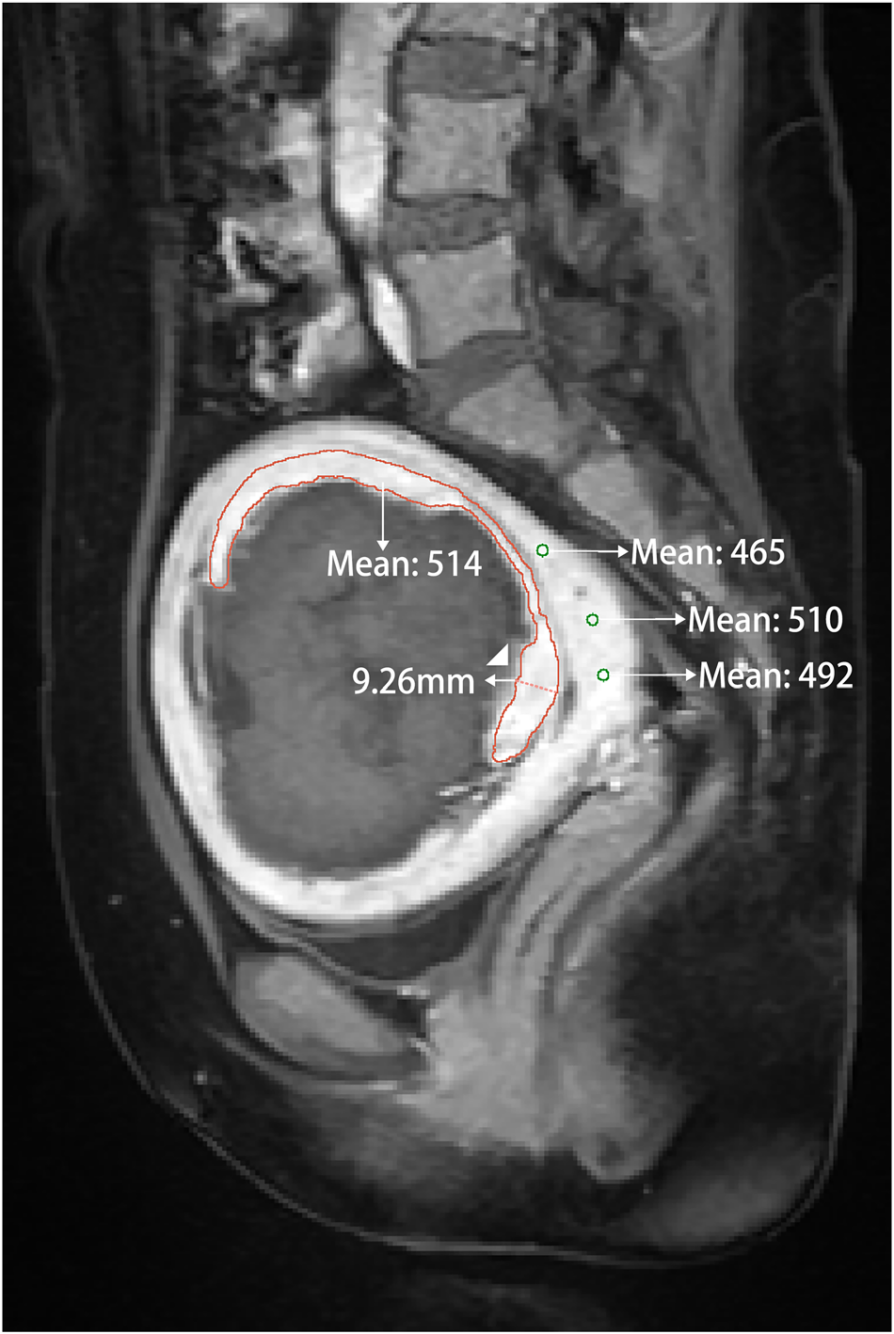
One day post-operation, ROI of the myometrium residual fibroid (RF) volume. Mean values: mean signal intensity values; ▲: RF thickness. ROI, region of interest

We used the open-source 3D-slicer software (Slicer 5.5.0) to outline the contours layer by layer on CE-MR images, measuring the residual fibroid volume (RFV) and Non-Perfusion Volume (NPV) on day 1 after treatment and 1 year later. Subsequently, by subtracting the RFV 1 year after RFV on day 1 after HIFU, the patients were divided into the RF regrowth group and the RF non-regrowth group. SI perfusion parameters were quantified using the RFV-myometrium and fibroid-myometrium SI ratios on CE-MRI on day 1 post-HIFU [20]. On CE-MRI slicers at the uterine artery phase around the 30s (sagittal position), three layers were continuously drawn along the edge of the RFV, and three positions of the myometrium were randomly drawn to obtain the average value automatically (Fig. 1). Before and after HIFU, the T2WI was used to draw three layers continuously along the edge of the uterine fibroids, and three positions of the myometrium were randomly drawn to obtain the average value automatically. When drawing the above, the ROI included the maximum area of the fibroid tissue to avoid partial volume effects.

Another evaluation was performed for the uterine position fibroid location, fibroid volume and number, FIGO classification, maximum fibroid diameter, RF thickness, and both preoperative and postoperative Funaki classification. The three types of fibroids, as classified by Funaki, are hypointense (SI lower than the iliacus muscle), isointense (SI lower than the myometrium but higher than the iliacus muscle), and hyperintense (SI higher than the myometrium) [21].

If there was a disagreement, the director of radiology department with 32 years of diagnostic experience made the final decision.

### Statistical analysis

Data analysis was performed using the SPSS software (version 27.0; IBM Corp., USA). Normally distributed data as mean ± standard deviation; skewed distribution data as median and interquartile range (IQR); and categorical data were expressed as numbers and percentages (%). In the univariate analysis, an independent sample *t*-test was used when the data were normally distributed and the variance was equal. The Mann–Whitney *U*-test was used when the data were not normally distributed, or the variance was unequal, and the chi-square test or Yates’ continuity correction method was used to compare the distribution of categorical data. We used Pearson’s or Spearman’s correlation analysis to analyze the correlation between two parameters. In the univariate analysis with significant differences, logistic regression analysis was adopted to determine the independent parameters predicting RF regrowth. A receiver operating characteristic curve (ROC) was drawn to analyze the predictive performance of the different parameters, and the area under the curve (AUC) was calculated [22]. Statistical significance was set at *p* < 0.05.

## Results

### Baseline characteristics of patients

A total of 164 patients were included in this study. Table 2 shows the baseline characteristics of the study population. The average age of the patients was 41 (35–45), and the median maximum diameter of uterine fibroids was 6.08 cm (4.78–7.5). The other baseline characteristics of the patients are shown in Supplementary Table 1.

**Table 2:** Comparison of baseline characteristics of RF regrowth and RF non-regrowth

Characteristic	Value (*n* = 164)	Regrowth (*n* = 91)	Non-regrowth (*n* = 73)	*p*-value
Clinical information				
Patient’s age (years)*	41.0 (35.0–45.0)	41.0 (35.0–44.0)	42.0 (35.0–46.0)	0.418
BMI*	22.37 (20.9–24.2)	22.1 (20.5–24.3)	22.58 ± 2.28	0.513
Gravidity*	3.0 (2.0–4.0)	3.0 (2.0–4.0)	3.0 (1.0–4.0)	0.799
Parity*	1.0 (0.0–1.0)	1.0 (0.0–1.0)	1.0 (0.0–1.0)	0.523
Age at menarche*	13.0 (12.0–14.0)	13.0 (12.0–14.0)	13.0 (12.0–13.0)	0.352
Menstruation*	5.5 (5.0–7.0)	5.0 (5.0–7.0)	6.0 (5.0–7.0)	0.925
Menstrual cycle*	28.0 (25.75–29.0)	28.0 (26.0–29.0)	28.0 (25.0–30.0)	0.871
Menstrual volume (N/L/H) (%)^‡^	95/8/61 (57.9/4.9/37.2)	51/4/36 (56.0/4.4/39.6)	44/4/25 (60.3/5.5/34.2)	0.767
Dysmenorrhea (Y/N) (%)^‡^	121/43 (73.8/26.2)	67/24 (73.6/26.4)	54/19 (74.0/26.0)	1
Menstrual regularity (R/O/D) (%)^‡^	137/18/9 (83.5/11.0/5.5)	74/11/6 (81.3/12.1/6.6)	63/7/3 (86.3/9.6/4.1)	0.668
Smoking and drinking (Y/N)^‡^	162/2 (98.8/1.2)	90/1 (98.9/1.1)	72/1 (98.6/1.4)	1
MRI information				
Uterine fibroid volume (cm^3^)*	76.31 (38.85–142.22)	82.69 (56.35–142.98)	57.64 (28.9–138.22)	**0.030**
RFV (cm^3^)*	13.08 (4.4–29.6)	19.57 (9.25–49.94)	6.94 (2.93–14.88)	**< 0.001**
NPV (cm^3^)*	54.3 (26.25–111.59)	55.47 (29.46–95.71)	53.03 (23.56–121.75)	0.631
NPVR (%)*	0.84 (0.67–0.92)	0.72 (0.54–0.85)	0.9 (0.83–0.94)	**< 0.001**
Number of fibroids*	3.0 (1.0–6.0)	3.0 (1.0–5.0)	2.0 (1.0–6.0)	0.564
Fibroids size (cm)*	6.08 (4.78–7.5)	6.29 (5.31–7.8)	5.79 ± 1.86	**0.010**
RF thickness (cm)*	5.48 (2.88–11.35)	10.2 (5.64–16.85)	3.07 (2.0–5.08)	**< 0.001**
FIGO classification (%)^‡^				**0.007**
0/1/2	4/8/6 (2.4/4.9/3.7)	4/5/1 (4.4/5.5/1.1)	0/3/5 (0/4.1/6.8)	
3/4/5	12/17/19 (7.3/10.4/11.6)	4/11/9 (4.4/12.1/9.9)	8/6/10 (11.0/8.2/13.7)	
6/7/8	33/8/9 (20.1/4.9/5.5)	13/5/8 (14.3/5.5/8.8)	20/3/1 (27.4/4.1/1.4)	
2-5/2-3	43/5 (26.2/3)	30/1 (33.0/1.1)	13/4 (17.8/5.5)	
Position of uterus (A/M/R) (%)^‡^	90/42/32 (54.9/25.6/19.5)	50/24/17 (54.926.4/18.7)	40/18/15 (54.8/24.7/20.5)	0.942
Location of fibroid (A/P/L/F/U/C) (%)^‡^	35/79/22/11/9/8 (21.3/48.2/13.4/6.7/5.5/4.9)	18/39/16/3/8/7 (19.8/42.9/17.6/3.3/8.8/7.7)	17/40/6/8/1/1 (23.3/54.8/8.2/11.0/1.4/1.4)	**0.010**
T2WI				
Pre Funaki classification (hypo/iso/hyperintensity)^‡^	12/123/27 (7.3/75/17.7)	4/59/28 (4.4/64.8/30.8)	1/64/8 (1.4/87.7/11)	**< 0.001**
Pre SI ratio of fibroid to myometrium*	0.56 (0.41–0.86)	0.7 (0.48–1.03)	0.44 (0.37–0.63)	**< 0.001**
Post Funaki classification (hypo/iso/hyperintensity)^‡^	6/121/37 (3.7/73.8/22.6)	1/61/29 (1.1/67/31.9)	5/60/8 (6.8/82.2/11)	**0.002**
Post SI ratio of fibroid to myometrium*	0.72 (0.57–0.96)	0.83 (0.65–1.03)	0.61 (0.53–0.77)	**< 0.001**
CE-MRI				
SI ratio of RF to myometrium^†^	1.05 ± 0.21	1.16 ± 0.18	0.88 (0.8–0.96)	**< 0.001**

Data are expressed as mean ± standard deviation, median (interquartile range), or *n* (%). Bold values indicate a significant difference (*p* < 0.05)

*BMI* body mass index (18.5–23.9 kg/m^2^), *N/L/H* normal/lightweight, *Y/N* yes/no, *R/O/D* regular/occasionally/disordered, *RFV* residual fibroid volume, *NPV(R)* non-perfused volume (ratio), *A/M/R* anteverted/mid position/retroverted, *A/P/L/F/U/C* anterior/posterior/lateral/fundus/uterine cavity/cervix, *SI* signal intensity, *CE* contrast enhancement

* Mann–Whitney *U*-test

† Independent two-sample *t*-test

‡ Chi-square tests (Pearson’s chi-square, continuous corrected chi-square)

We compared the characteristics of the two groups of patients. There were no statistically significant differences (*p* > 0.05) in patient age, BMI, parity, menstrual period and cycle, menstrual volume, dysmenorrhea, menstrual regularity, smoking and drinking history, uterine position, NPV, or number of fibroids (Table 2). There were significant differences in the volume and position of the uterine fibroids, RFV, residual fibroid (RF) thickness, NPVR, FIGO classification, CE-MRI RF-myometrium SI ratio on day 1 after treatment, preoperative and postoperative T2WI fibroid-myometrium SI ratio, and Funaki classification between the regrowth group and non-regrowth group (*p* < 0.05) (Table 2).

### Results of multivariate and correlational analysis

The data in this study were suitable for logistic regression analysis, and we set the RF regrowth as the dependent variable. We performed multivariate binary logistic regression on the variables with the above statistical differences. The results showed that the independent factors were RF thickness (*p* < 0.001) and the CE-MRI RF-myometrium SI ratio (*p* < 0.001) (Table 3).

**Table 3:** Binary logistic regression analysis results of independent factors affecting RF regrowth

Independent factors	B	SE	Wald	df	Sig.	Exp (B)	95% Cl for EXP (B)	
							Lower	Upper
SI ratio of RF to myometrium	0.935	0.175	28.669	1	**< 0.001**	2.548	1.809	3.588
RF thickness (mm)	0.341	0.079	18.773	1	**< 0.001**	1.406	1.205	1.641
Constant	−11.55	1.967	34.473	1	**< 0.001**	-	-	

Bold values indicate significant differences (*p* < 0.05)

*RF* residual fibroid, *SI* signal intensity, *CE* contrast enhancement

**Fig. 2:**
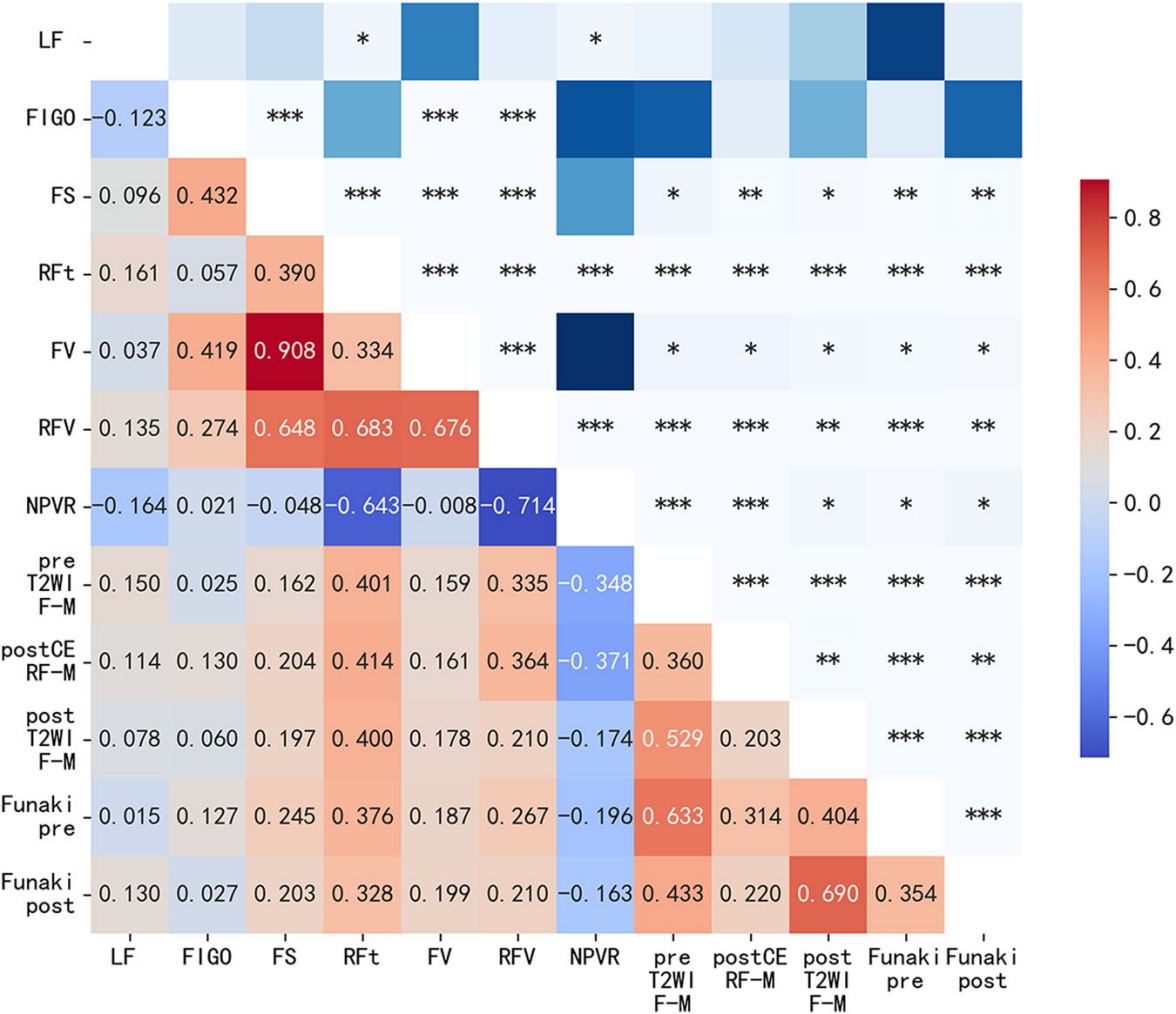
Heatmap of correlations between different variables. LF, location of fibroid; FS, fibroid size; RFt, residual fibroid thickness; FV, fibroid volume; RFV, residual fibroid volume; NPVR, non-perfused volume ratio; pre/post-T2WI F-M, pre/postoperative SI ratio of fibroid to myometrium; post CE RF-M, postoperative SI ratio of RF to myometrium. *p < 0.05; **p < 0.01; ***p < 0.001

**Fig. 3:**
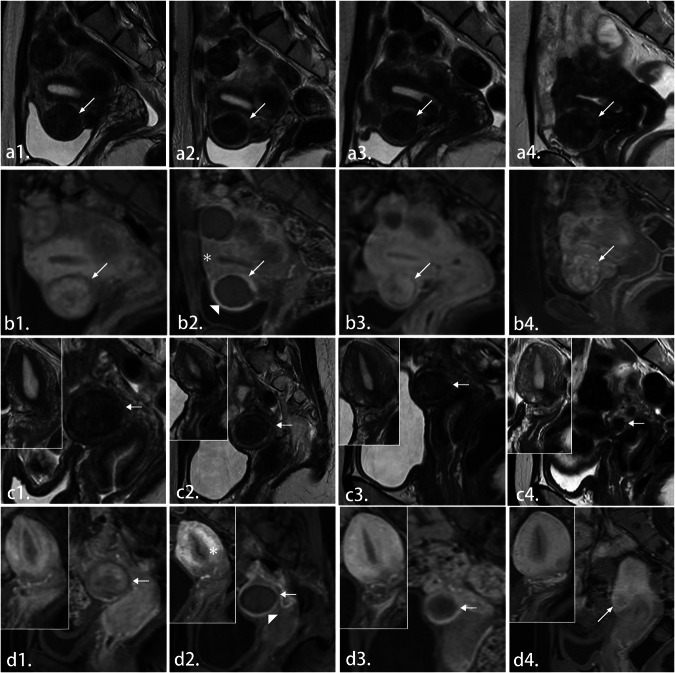
From left to right are MR images of two uterine fibroid patients in the RF regrowth group and non-regrowth group before HIFU treatment, 1 day after surgery, 6 months, and 1 year. Among them, **a** and **b** are T2WI and CE-MRIs of the same patient, and **c** and **d** are T2WI and CE-MRIs of another patient. ↑: Uterine fibroids; ▲: RF; *: Uterine muscle layer. **a1**–**a4** For hypointense fibroids, the SI is relatively low, and the signal inside the fibroid is uniform. **b1**–**b4** In the arterial phase, 30 s after the injection of the CE-MRI contrast agent, the signal intensity (SI) is elevated. One day after treatment, the signal around the RF was significantly stronger than that of the myometrium. One year later, the NPV was not visible, and the fibroid was slightly larger than before the operation. **c1**–**c4** For hypointense fibroids, the SI is relatively low, and the fibroid signal is slightly uniform. **d1**–**d4** In the arterial phase, 30 s after injection of the CE-MRI contrast agent, the SI was elevated. One day after the treatment, the signal around the RF was significantly lower than that of the myometrium. One year later, the fibroid was significantly reduced and smaller than before the operation

**Fig. 4:**
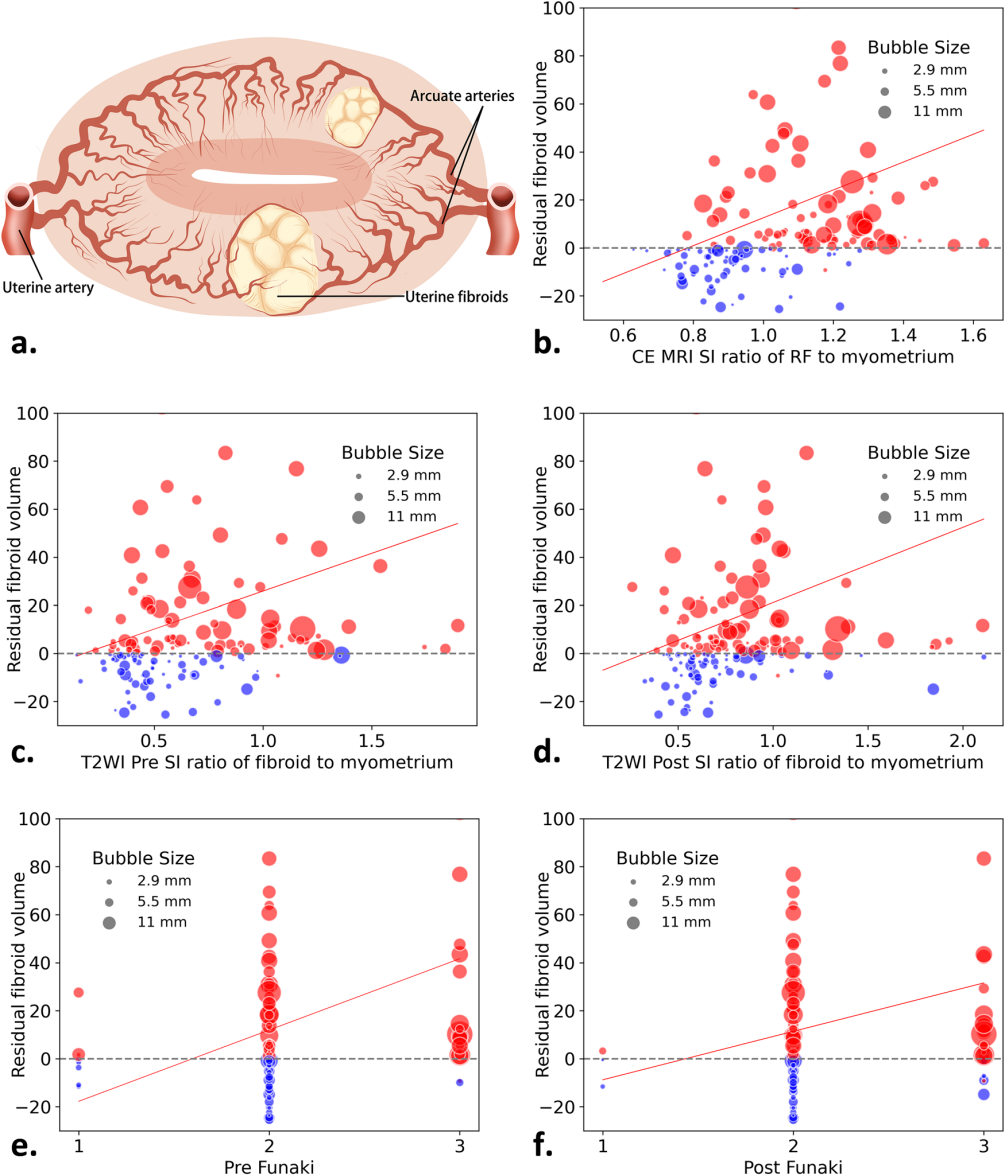
Scatter plot trends in T2WI and CE-MRI RF regrowth and non-regrowth groups. The horizontal axis of the image represents the SI value of fibroid-myometrium and Funaki classification. In contrast, the vertical axis represents the increase or decrease in RF volume 1 year after treatment. The size of the scatter points represents the thickness of RF 1 day after treatment. The diagonal line represents the trend of RF enlargement or shrinkage. Red represents the RF regrowth group, and blue represents the RF non-regrowth group. **a** Schematic diagram of the blood supply in the cross-section of the uterine fibroid. The larger fibroid at the bottom is bilaterally supplied by the left and right uterine arteries extending to the myometrium, while a single uterine artery supplies the fibroid at the top. **b** The predicted trend of RF-myometrium ratio 1 day after treatment. **c** Predicted trend of preoperative T2WI fibroid-myometrium ratio. **d** Predicted trend of T2WI fibroid-myometrium ratio 1 day after treatment. **e** Predicted trend of preoperative Funaki classification. **f** Predicted trend of postoperative Funaki classification

To further reflect the relationship between the two independent factors of CE-MRI RF-myometrium SI ratio and RF thickness and other variables that affected fibroid regrowth in the past, we used Spearman’s correlation analysis to compare the correlation between variables with significant differences (Fig. 2). Based on CE-MRI and T2WI, the images of RF regrowth 1 year after different classifications are shown in Fig. 3. We created a scatter plot to reflect the volume changes in the RFs and the association between the different sequence SI ratios and RF thickness (Fig. 4). After correlation analysis, CE-MRI SI ratio found to be positively correlated with an increased RFV (*r* = 0.24, *p* < 0.002).

### Changes in the volume of uterine fibroids post-HIFU treatment

**Fig. 5:**
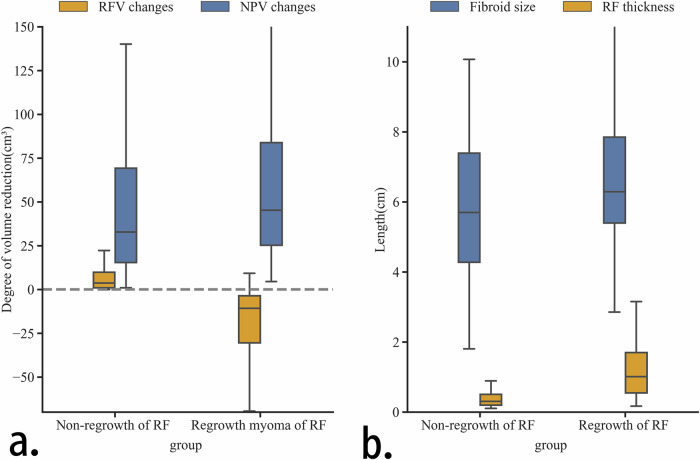
**a** The reduction in volume of RF and NPV 1 year later. **b** The size of the fibroid and the RF thickness 1 day after treatment

We separately calculated the volume changes in the RFV and NPV of the uterine fibroids over 1 year. In the regrowth group, the volume of fibroids decreased by 24.15 (7.64–61.34) cm^3^, while in the non-regrowth group, it decreased by 39.73 (19.06–74.13) cm^3^. We found that the NPV decreased more in the regrowth group than in the non-regrowth group (Fig. 5). Specifically, the NPV in the regrowth group decreased by 44.92 (25.27–83.09) cm^3^, compared to 32.82 (15.41–69.24) cm^3^ in the non-regrowth group (*p* < 0.048). Additionally, in the regrowth group, the increase in RFs was negatively correlated with the decrease in NPV (*r* = −0.496, *p* < 0.001), whereas in the non-regrowth group, the decrease in RFs was positively correlated with the decrease in NPV (*r* = −0.518, *p* < 0.001).

### Comparing CE-MRI and T2WI in predicting RF regrowth

**Fig. 6:**
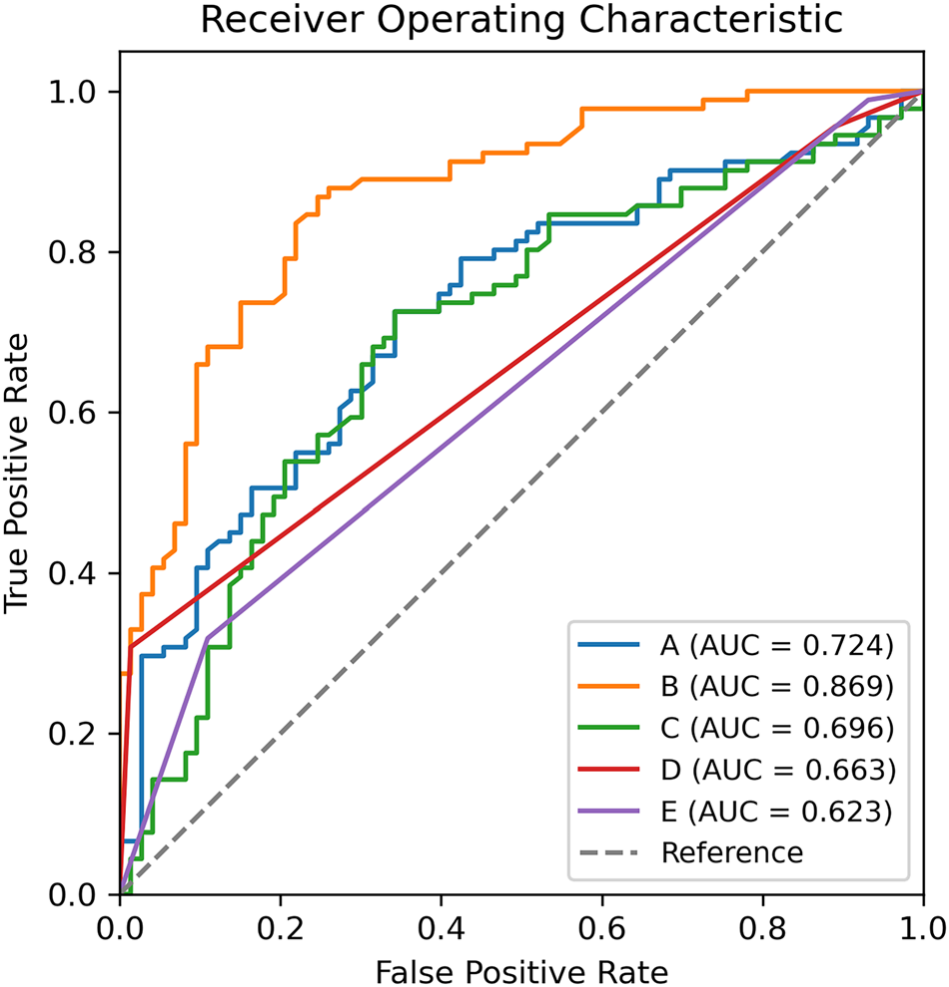
ROC curves predicting RF regrowth using the SI ratio of T2WI and CE-MRI. AUC, area under the curve. A: Preoperative T2WI fibroid-myometrium SI ratio; B: Postoperative day 1 CE-MRI RF-myometrium SI ratio; C: Postoperative day 1 T2WI fibroid-myometrium SI ratio; D: Preoperative Funaki classification; E: Postoperative Funaki classification

We analyzed the postoperative 1-day CE-MRI RF-myometrium SI ratio, preoperative and postoperative T2WI fibroid-myometrium SI ratios, and Funaki classification by ROC curve (Fig. 6). Then we found that for predicting RF regrowth, the RF-myometrium SI ratio (AUC = 0.869, 95% CI: 0.814–0.922) was superior to the preoperative and postoperative T2WI fibroid-myometrium SI ratio (AUC = 0.724, 95% CI: 0.645–0.801), (AUC = 0.696, 95% CI: 0.611–0.782), and to preoperative and postoperative Funaki classification (AUC = 0.663, 95% CI: 0.606–0.716), (AUC = 0.623, 95% CI: 0.562–0.683). The cutoff value of CE-MRI RF-myometrium SI ratio is 0.968.

### External validation

We retrospectively collected from 30 patients treated at another hospital in 2017 as our external validation group. There were no statistically significant differences (*p* < 0.05) in baseline characteristics between the internal and external validation groups (Supplementary Table 1).

In the external validation set, the RF regrowth CE-MRI RF-myometrium SI ratio (AUC = 0.915) similarly showed superior predictive accuracy compared to the preoperative and postoperative T2WI fibroid-myometrium SI ratios (AUC = 0.833, AUC = 0.86) and the preoperative and postoperative Funaki classifications (AUC = 0.797, AUC = 0.752) (Supplementary Fig. 1).

### Long-term follow-up

Among all the 164 participants, 149 were followed up. The median follow-up was 26 (24–30) months. Fifty-one patients underwent re-intervention; 88% of them exhibited RF regrowth within a year. Among 85 patients from the RF regrowth group, 45 (52.9%) underwent re-interventions, 18 (21.2%) had persistent symptoms, 16 (18.8%) experienced symptom improvement, and only 6 (7.1%) had fibroid shrinkage. In the RF non-regrowth group, only 6 patients (9.4%) had re-interventions, 7 (11%) had persistent or worsening symptoms, 34 (53.1%) had symptom improvement, and 17 (26.6%) had fibroid shrinkage. There were significant statistical differences in re-intervention rate, persistence or improvement of symptoms, and changes in fibroid volume (*p* < 0.001).

Spearman correlation analysis revealed significant positive correlations between the number of re-interventions and the increase in RF volume (*r* = 0.455, *p* < 0.001), as well as between the number of re-interventions and the SI ratio of RF-myometrium (*r* = 0.268, *p* < 0.001).

## Discussion

The different blood supplies of residual fibroid (RF) can affect treatment outcomes and long-term prognosis. Currently, few studies focus on the predictability of CE-MRI RF-myometrium SI ratio for the RF after HIFU treatment.

Univariate analysis showed differences in fibroid diameter, volume, RFV, NPVR, and fibroid position within the uterus between the regrowth and non-regrowth groups. Our finding that the cutoff value of NPVR was 76% is consistent with the results of previous research [23, 24]. The higher the NPVR is, the less chance of RF to regrow and require re-intervention after HIFU ablation [11]. Previous studies have shown that patient age is an essential factor affecting fibroid regeneration [25]. Younger patients are more likely to need re-intervention, but it is also believed that NPVR ≥ 80% is an effective measure to reduce re-intervention in young patients [26]. In our study, there was no difference in age between the two groups. A possible reason for this is that the median NPVR of the RF non-regrowth group reached 90%, so this factor was effectively eliminated.

In FIGO classification, significant differences in RF regrowth were found between the groups of type 2–5 and cervical fibroids. Their RFs, with SI ratios of 1.1 (0.949–1.22) for type 2–5 and 1.184 (0.944–1.28) for cervical, were more prone to regrowth, as indicated by higher CE-MRI SI ratios. Therefore, we speculated that these two FIGO types have a richer blood supply compared to others.

RF thickness and RF-myometrium SI ratio of CE-MRI are independent factors affecting RF regrowth and are related to multiple indicators. Post-treatment RF thickness affects fibroid regrowth [7, 8]. It was positively correlated with CE-MRI RF-myometrium SI ratio. Therefore, we speculated that certain fibroids with rich blood supply can be difficult to ablate and prone to regrowth. In the regrowth group, the increase in RFV of the fibroids was negatively correlated with the reduction in NPV, while in the non-regrowth group, RFV was positively correlated with the reduction in NPV. This may suggest that the fibroids in the regrowth group are surrounded by a relatively richer blood supply.

The CE-MRI of uterine fibroids is closely related to their histopathological blood supply. When the RF SI is higher than that of the myometrium SI during the arterial phase, it means that RF has more blood supply than the myometrium, indicating maybe more than one supplying artery or even uncommonly vascular variations [13]. DSA shows that the blood supply to the uterine fibroids is different [10, 14, 27, 28]. We speculate that variations in uterine fibroid blood vessels affect the order of perfusion; therefore, RFs may also have different or multiple blood supplies. The RF exhibited a higher SI than the myometrium, indicating more abundant blood supply. If the ratio of RF-myometrium is higher than the cutoff value of 0.968, the RF is likely to regrow, while those below this value may shrink or even disappear.

Preoperative T2WI and CE-MRI in the treatment of fibroid regrowth have been widely explored in previous studies [8, 11, 18, 28]. The T2WI was thought to reflect the composition of the fibroid [20, 21, 29–33]. It is generally believed that hyperintense fibroids on the T2WI and high enhancement on CE-MRI are prone to regrowth, with the latter being an independent factor for fibroid regrowth, as confirmed by our research [16, 34, 35]. In our study, there were 123 cases (75%) of isointense fibroids before treatment, of which 59 cases (64.8%) had fibroids regrowth. However, the Funaki classification could not predict the regrowth of isointense fibroids owing to its visual inspection. Our results showed that the CE-MRI SI ratio 1 day after treatment could be a better predictor than the Funaki classification. We comprehensively compared the Funaki classification and fibroid-myometrium SI ratio of the T2WI before and after HIFU treatment with the CE-MRI RF-myometrium SI ratio. Observing the RFs through CE-MRI other than the entire fibroid through T2WI after HIFU treatment can more accurately indicate the regrowth of the RF.

Radiomics is a technique for extracting high-throughput data [36]. Previous studies have used radiomics to predict the RF regrowth through T2WI [7, 37, 38]. However, extracting image data requires complex post-processing support [39]. Our study found that the blood supply of RFs with higher ratios has multiple blood supplies and is more prone to regrowth through the CE-MRI RF-myometrium SI ratio, and the long-term prognosis can be evaluated 1 day after treatment. This may also help solve the problem that follow-up usually depends on MRI examination, which is more convenient and economical.

It has been reported that fibroid initially decreases in volume significantly after HIFU treatment [17]. We found that in the RF regrowth group, only 14 (15.4%) of 91 patients had enlarged fibroids after 1 year. However, the remaining 77 (84.6%) patients experienced a significant increase in RF volume, and the RF regrowth group exhibited a higher SI ratio. Long-term follow-up revealed a significant positive correlation among RF regrowth, the SI ratio of RF-myometrium, and the number of re-interventions, with 45 (52.9%) of patients in the RF regrowth group requiring additional treatment. Therefore, the CE-MRI RF-myometrium SI ratio can reflect the re-intervention for a long term.

To validate the predictive ability of the CE RF-myometrium SI ratio, we conducted an external validation. Then we found results showed that the higher the RF-myometrium ratio is, the more likely it is to regrow quickly. By using ROC curve analysis, the CE-MRI RF-myometrium SI ratio can be a better predictor in the external validation group than in the internal validation group. However, it generally reflects the trend of the RF-myometrium ratio in predicting RF regrowth. Because of the small sample size of the external validation group and the complex factors for RF regrowth, this finding still needs to be further confirmed. Nevertheless, in predicting regrowth, the RF-myometrium ratio was more accurate and reliable than the preoperative and postoperative T2WI and the Funaki classification.

Our study had limitations. All the data of this retrospective study were obtained only from two medical centers. More data from multicenter studies with larger sample size are still needed to confirm our findings. In addition, other quantitative parameters, such as the time-intensity curve of DCE-MRI, could be introduced in this analysis for better results. So far, the relationship between the CE-MRI SI ratio, which reflects RF blood supply characteristics, and its regrowth provides a more preferable reference for clinical practice.

## Conclusion

Compared to the Funaki classification and SI ratio of the T2WI, the CE-MRI RF-myometrium SI ratio could reliably and accurately predict RF regrowth by reflecting the blood supply within the myometrium. For cases of uterine fibroids after HIFU treatment, our study provides a theoretical basis for the clinical evaluation of long-term recovery and further treatment plans.

## Supplementary information

ELECTRONIC SUPPLEMENTARY MATERIAL

## Data Availability

The data that support the findings of this study are available from the corresponding author, F.J.L., upon reasonable request.

## Abbreviations

AUC: Area under curve
CE-MRI: Contrast-enhanced magnetic resonance imaging
HIFU: High-intensity focused ultrasound
NPV: Non-perfused volume
NPVR: Non-perfusion volume ratio
RF: Residual fibroid
RFV: Residual fibroid volume
ROC: Receiver operating characteristic curve
SI: Signal intensity
T2WI: T2-weighted imaging
