# Supplementary material for: Predictive value of contrast-enhanced MRI for the regrowth of residual uterine fibroids after high-intensity focused ultrasound treatment
Source: Insights Imaging. 2024 Nov 15;15:274. doi: 10.1186/s13244-024-01839-w (PMC11568090; doi:10.1186/s13244-024-01839-w)
Supplement: Supplementary file 1 — ELECTRONIC SUPPLEMENTARY MATERIAL [file 13244_2024_1839_MOESM1_ESM.pdf]

Predictive Value of Contrast-Enhanced MRI for the Regrowth of Residual Uterine Fibroids After High Intensity Focused Ultrasound Treatment

ELECTRONIC SUPPLEMENTARY MATERIAL

Supplementary Table 1. Comparison of baseline characteristics between the internal and external validation group of patients

Characteristic	Internal group (n=164)	External validation (n=30)	*p*-Value
Patient's age (years) *	90/42/32(54.9/25.6/19.5)	15/6/9(50.0/20.0/30.0)	0.608
BMI*	22.37(20.9 - 24.2)	22.02 ± 3.06	0.061
Gravidity*	3.0(2.0 - 4.0)	2.0(1.0 - 3.0)	0.147
Parity*	1.0(0.0 - 1.0)	1.0(0.0 - 1.0)	0.063
Age at menarche*	13.0(12.0 - 14.0)	13.0(12.0 - 13.0)	0.082
Menstruation*	5.5(5.0 - 7.0)	5.93 ± 1.34	0.244
Menstrual cycle*	28.0(25.75 - 29.0)	29.0(25.5 - 31.0)	0.11
Menstrual volume (N/L/H) (%) ‡	95/8/61(57.9/4.9/37.2)	12/6/12(40.0/20.0/40.0)	0.565
Dysmenorrhea(Y/N) (%) ‡	125/39(76.2/23.8)	22/8(73.3/26.7)	0.568
Menstrual regularity(R/O/D) (%) ‡	141/15/8(86.0/9.1/4.9)	26/2/2(86.7/6.7/6.7)	0.316
Smoking and drinking(Y/N) (%) ‡	162/2(98.8/1.2)	29/1(96.7/3.3)	0.852
Uterine fibroid volume (cm^3)*	76.31(38.85 - 142.22)	48.76(24.34 - 97.01)	0.068
RFV (cm^3)*	13.08(4.4 - 29.6)	8.6(4.99 - 19.3)	0.419
NPV (cm^3)*	54.3(26.25 - 111.59)	39.09(16.58 - 82.14)	0.292
NPVR (%)*	0.84(0.67 - 0.92)	0.83(0.69 - 0.89)	0.111
Number of fibroids*	3.0(1.0 - 6.0)	2.0(1.0 - 3.0)	0.914
Fibroids size(cm)*	6.08(4.78 - 7.5)	5.24(4.48 - 6.73)	0.347
RF thickness(cm) *	5.48(2.88 - 11.35)	8.52(5.16 - 14.18)	0.954
FIGO classification (%) ‡	4/8/6/12/17/19/33/8/9/43/5(2.4/4.9/3.7/7.3/10.4/11.6/20.1/4.9/5.5/26.2/3.0)	4/8/6/6/4/4/6/1/1/7/2(2.4/4.9/3.7/20.0/13.3/13.3/20.0/3.3/3.3/23.3/6.7)	0.845
Position of uterus (A/M/R) (%) ‡	90/42/31(54.9/25.6/19.5)	15/9/6(50/30/20)	0.385
Location of fibroid (A/P/L/F/U/C) (%) ‡	35/79/22/11/9/8(21.3/48.2/13.4/6.7/5.5/4.9)	4/13/7/4/1/1(13.3/43.3/23.3/13.3/3.3/3.3)	**0.008**
T2WI			

Pre Funaki classification (hypo/iso/hyperintensity) ‡	12/123/29(7.3/75.0/17.7)	2/17/11(6.7/56.7/36.7)	0.053
Pre SI ratio of fibroid to myometrium*	0.56(0.41 - 0.86)	0.55(0.45 - 0.67)	0.257
Post Funaki classification (hypo/iso/hyperintensity) ‡	6/121/37(3.7/73.8/22.6)	0/21/9(0/70.0/30.0)	0.342
Post SI ratio of fibroid to myometrium*	0.72(0.57 - 0.96)	0.86±0.22	0.111
CE-MRI			
SI ratio of RF to myometrium †	1.05±0.21	1.09±0.11	0.134

Note: BMI: body mass index（18.5-23.9kg/m^2）; N/L/H: Normal/Light/Heavy; R/O/D: Regular/Occasionally/Disordered; RF(V): residual fibroid (volume); NPV(R): Non-perfused volume (ratio); SI: signal intensity; A/M/R: Anteverted/Mid position/Retroverted; A/P/L/F/U/C: Anterior/Posterior/Lateral/Fundus/Uterine cavity/Cervix; CE: contrast enhancement. Date expressed as mean ± standard deviation, median (interquartile range), or n (%). * Mann-Whitney U-test; ‡ Chi-square tests (Pearson's chi-square, Continuous corrected chi-square); † Independent two-sample t-test. Bold values indicate a significant difference ($p < 0.05$).

Supplementary Figure 1. ROC curves predicting RF regrowth using the SI ratio of T2WI and CE-MRI in an external validation cohort.

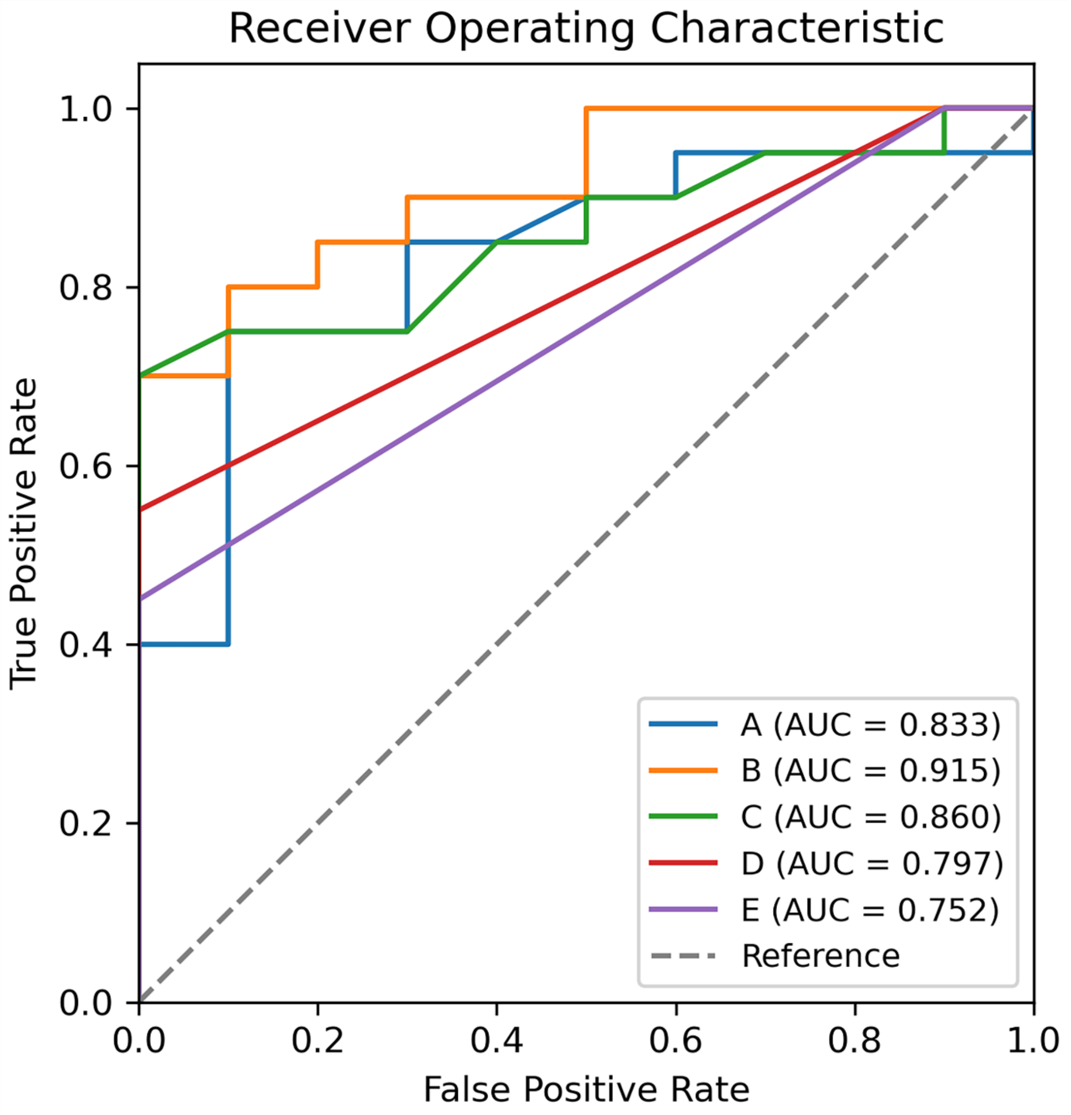

Note: AUC, area under the curve; A: Preoperative T2WI fibroid-myometrium SI ratio (AUC = 0.833, 95% CI: 0.655-0.971); B: Postoperative day 1 CE MRI RF-myometrium SI ratio (AUC = 0.915, 95% CI: 0.804-0.995); C: Postoperative day 1 T2WI fibroid-myometrium SI ratio (AUC = 0.860, 95% CI: 0.702-0.971); D: Preoperative Funaki classification (AUC = 0.797, 95% CI: 0.682-0.894); E: Postoperative Funaki classification (AUC = 0.752, 95% CI: 0.625-0.864).
